# HIV Pre‐Exposure Prophylaxis (PrEP) Users' Experiences of PrEP Access, Sexual Behaviour, and Well‐Being During the COVID‐19 Pandemic: A Welsh Qualitative Study

**DOI:** 10.1111/hex.70064

**Published:** 2024-10-17

**Authors:** Zahraa Khammas, David Gillespie, Adam Dale Newman Williams, Jane Nicholls, Fiona Wood

**Affiliations:** ^1^ Cardiff University School of Medicine Cardiff University Cardiff UK; ^2^ Centre for Trials Research Cardiff University Cardiff; ^3^ Division of Population Medicine, PRIME Centre Wales Cardiff University Cardiff UK

**Keywords:** COVID‐19, HIV, PrEP, qualitative research, sexual behaviour

## Abstract

**Background:**

HIV Pre‐Exposure Prophylaxis (PrEP) has been available in Wales since 2017. The coronavirus disease (COVID‐19) pandemic impacted UK sexual health services, leading to a reduction in service provision. There is a lack of research on the experiences of PrEP users during this time.

**Objective:**

We aimed to explore the experiences of PrEP users in Wales following the introduction of COVID‐19 pandemic measures.

**Methods:**

We conducted a secondary data analysis of two prior interview studies (DO‐PrEP and UPrEP). Data collection was undertaken between May 2020 and February 2021 using remote interviewing. Semi‐structured interviews were conducted. Participants were ≥18 years of age, residents of Wales, current or previous PrEP users, and men who have sex with men (MSM). Reflexive thematic analysis was conducted.

**Results:**

A total of 32 interviews were included in the analysis. Themes include the following: (1) PrEP use during COVID‐19, (2) sexual behaviour and relationship changes following COVID‐19 restrictions, (3) NHS service provision during the COVID‐19 pandemic, and (4) wider contextual effects of the pandemic. Participants reported a change in PrEP use (pausing or switching to event‐based PrEP). Participants reported reduced access to clinics and appointments. Support for changing to event‐based PrEP was varied. Social isolation resulted in varied responses to lockdown rules, especially in later lockdowns.

**Conclusion:**

This study provides patient perspectives on the challenges the COVID‐19 pandemic posed to PrEP use and access. It offers insights into the broader support needs around PrEP use when an individual's circumstances change. Flexible models of PrEP provision, which can be adapted to the patient's needs, will be essential as PrEP delivery extends into the wider community.

**Patient and Public Contribution:**

Both the DO‐PrEP and UPrEP studies received input from various stakeholders in the design of the topic guides for the study; these included public lay members, PrEP users, PrEP providers, and individuals involved with HIV advocacy and policy.

## 1 Introduction

Human immunodeficiency virus (HIV) Pre‐Exposure Prophylaxis (PrEP) is taken by HIV‐negative people and can be taken daily or event‐based (the latter regimen is only available for men who have sex with men [MSM]) [1]. The World Health Organisation (WHO) released guidance in 2012 and 2016 on PrEP use for individuals who fall under the categories that include MSM, people in prisons and closed settings, people who inject drugs, sex workers and transgender people [2, 3]. PrEP is highly effective (99%) when taken as instructed. The coronavirus disease (COVID‐19) pandemic had a substantial impact on sexual health services [4]. In the United Kingdom, there was a loss of access to in‐person care, meaning that PrEP and sexually transmitted infection (STI) testing services were impacted. Remote consultations were introduced, and only individuals triaged as high priority or urgent were still seen face to face [4]. There had been a general decline in the number of new HIV diagnoses in the United Kingdom until 2020 [5]. In the five years before the COVID‐19 pandemic, the number of people diagnosed with HIV in Wales usually ranged between 90 and 150 each year, and around half of these individuals were MSM [6] and that has remained fairly consistent post‐COVID with 101 new diagnoses of HIV in Wales in 2022 [7].

In Wales, PrEP has been available since 2017 for those who are deemed to benefit from PrEP use, for example, people who engage in sexual behaviours such as condomless sex [8]. In May 2020, Public Health Wales (PHW) expanded their postal testing service, test and post, where STI test kits could be ordered through the Sexual Health Wales (SHW) website, formerly known as Frisky Wales [9]. This service was rolled out in May 2020 in response to service restrictions.

Studies have shown that the introduction of social distancing measures results in a reduction in sexual activity in MSM, a reduction in PrEP use and reduced coverage of condomless sex by PrEP [10, 11, 12]. However, there is a lack of qualitative research on PrEP users' experiences during the COVID‐19 pandemic [13]. This study aims to explore the experiences of PrEP users in Wales following the introduction of COVID‐19 pandemic measures. Specifically, we explore PrEP use, access to PrEP, sexual behaviours, and general well‐being among MSM during the COVID‐19 pandemic.

## 2 Methods

### 2.1 Data Sets and Study Design

The data for this secondary analysis are taken from two prior studies: DO‐PrEP and UPrEP. DO‐PrEP explored PrEP adherence and sexual behaviour in MSM accessing PrEP through sexual health clinics in Wales [14]. UPrEP focuses on the perceived relationship between PrEP, STIs, and antimicrobial resistance in Wales among MSM [15].

Both studies used qualitative semi‐structured interviews. The studies were designed pre‐COVID‐19, and the interview schedule did not specifically include COVID‐19 as a point of discussion. However, as data collection occurred during the pandemic, the impact of COVID‐19 was raised during discussions.

### 2.2 Participant Recruitment

Participants for DO‐PrEP (*n* = 21) were known to be receiving PrEP through NHS sexual health clinics in Wales and were recruited from the existing pool of participants within a larger study [16]. Participants for UPrEP (*n* = 20) were recruited using advertisements on social media platforms followed by snowball sampling. Recruitment aimed to ensure a stratified sample of participants (prior PrEP use, current PrEP use, and no experience of PrEP use [transcripts from this latter category were removed from the analysis of this secondary analysis study]). The inclusion and exclusion criteria for both studies are outlined in Table 1.

**Table 1:** Inclusion and exclusion criteria of DO‐PrEP and UPrEP.

Study	Inclusion criteria	Exclusion criteria
DO‐PrEP	Men who have sex with men (MSM)	Lacks the capacity to give consent.
	> 18 years old	Unable or unwilling to provide a mobile telephone number.
	Attending an NHS sexual health clinic in Wales for HIV Pre‐Exposure Prophylaxis	Does not have a smartphone or mobile telephone with access to the Internet.
		Unable or unwilling to provide a valid email address.
		Unable or unwilling to use the Medication Monitoring System (MEMS) for the duration of study.
UPrEP	MSM	Lacks the capacity to give consent.
	> 18 years old	Unable to converse in English.
	Currently living in Wales	No access to electronic devices that enabled Zoom.

### 2.3 Data Collection

All participants took part in one‐to‐one interviews on an online video platform, Zoom. Participants were given a unique meeting ID and password. Informed verbal consent was obtained and audio recorded in a separate file at the beginning of each interview. DO‐PrEP interviews took place between May 2020 and November 2020, whereas UPrEP interviews took place between September 2020 and February 2021. Therefore, data collection took place during official ‘lockdown’ periods in which movement is restricted in the interests of public safety, during ‘firebreaker lockdowns’ which are short lockdowns of around 2 weeks designed to alleviate pressure on health services and during other periods of general restrictions on social mixing. Both studies involved participants from across Wales. Topic guides were reviewed by stakeholder groups for each study to collaboratively refine it, ensuring it was relevant and appropriate. The stakeholder groups included people from various groups such as PrEP users, PrEP providers and those involved in HIV advocacy and policy. Interviews were audio recorded and transcribed verbatim by a professional transcription service.

### 2.4 Data Analysis

Secondary data analysis was performed on transcripts where COVID‐19 had been discussed during the interviews, and the participants had ever used PrEP (19 participants from DO‐PrEP and 13 participants from UPrEP) using reflexive thematic analysis [17]. Braun and Clarke outline the process of thematic analysis, and more recently reflective thematic analysis as six stages: familiarising yourself with the data set, labelling the data with words/phrases which will aid in answering the research question (coding), creating the initial themes, developing and regular reviewing of themes, finalising the themes and writing up the results [17]. Reflexivity allowed the authors to use their experiences, beliefs and background in data analysis. NVivo version 12 was used to aid the analytical process. An initial coding framework was developed using four interview transcripts by the lead author. Following this, all co‐authors independently coded two transcripts each. All analysts then met to redefine the coding framework. This was then used to code the remaining data.

### 2.5 Reflexivity and the Research Team

The lead author is a 22‐year‐old female, British‐Arab, cisgender heterosexual. Co‐authors are a mix of ages (25–56 years), sexualities and genders. Authors are from a variety of disciplinary backgrounds: sociology, psychology, clinical medicine specialising in sexual health, and trials research. All authors have an academic interest in infectious diseases and sexual health, and two authors have a clinical role. Regular team meetings were held to protect against any unconscious bias.

### 2.6 Ethical Approvals

A favourable ethical opinion was given by the NHS Wales Research Ethics Committee for DO‐PrEP and Cardiff University's School of Medicine Research Ethics Committee for UPrEP. The individual (Z.K.) who undertook the secondary analysis did not have access to participant‐identifiable data. This individual was named on the study delegation log as having access to the research data and only accessed those interview transcripts via secure university computer networks. The team worked in accordance with recommendations from the People First Charter HIV terminology document [18] to ensure that appropriate language and understanding of sexual behaviours among the LGBTQ+ community were used during the design, data collection, data analysis, and writing stages of the study. We were also mindful that during lockdowns, there were restrictions put in place with people being fined if found to be in breach of those conditions. We stressed to participants the confidential nature of the information that people shared and the voluntary nature of that sharing.

### 2.7 Patient and Public Contribution

DO‐PrEP involved several groups in creating the research materials for the study such as PrEP users, PrEP providers and individuals involved with HIV advocacy and policy. UPrEP research materials were informed by a small stakeholder group including public members, clinicians and HIV advocacy group members. These individuals assisted with the formation of the interview schedules and participant‐facing materials; however, there were no changes made to the schedules following PPI feedback related to the COVID‐19 pandemic.

## 3 Results

Nineteen of the 21 interviews were included in the analysis from DO‐PrEP and 13 of the 20 interviews from UPrEP (Table 2) resulting in 32 interviews available for secondary analysis. All participants were cisgender men, with the vast majority being white British men. Most of them were educated to a degree level or equivalent. The age range of participants was 20–53 years.

**Table 2:** Participant demographics for the secondary analysis data set.

Variables		DO‐PrEP (*N* = 19)		UPrEP (*N* = 13)	
		Frequency	%	Frequency	% (*n*)
Sex	Male	19	100	13	100
Gender identity	Cisgender	19	100	13	100
Ethnicity	White British	18	94.7	12	92.3
	White European	1	5.3	1	7.7
Education level	Educated to degree level or equivalent	11	57.9	12	92.3
	Completing undergraduate degree	0		1	7.7
	Educated to A‐levels or equivalent	7	36.9	0	0
	Educated to GCSE level (A*–C grades) or equivalent	1	5.3	0	0
PrEP status at recruitment	Starting PrEP for the first time (at recruitment)	4	21.1	0	0.0
	Previously used PrEP/current PrEP user	15	78.9	13	100
Relationship status	Single	16	84.2	7	53.8
	In a relationship	2	10.5	6	46.2
	Married	1	5.3	0	0.0
Age		Mean = 34		Mean = 27	
		Range = 22–53		Range = 20–53	
		SD = 8.95		SD = 9.53	

Several initial codes were developed, which were then organised into sub‐themes and four major themes as illustrated in Table 3. Each of the main themes will be described with example quotes from across the data sets.

**Table 3:** Themes, sub‐themes, and coding.

Themes	Subthemes	Initial coding
1. PrEP use during the COVID‐19 pandemic	Stopping and re‐starting PrEP	Changes to dosing. Continued PrEP as normal.
	Adopted flexible approaches to PrEP, i.e., event‐based dosing	Feelings to changes. Obtained PrEP from elsewhere. Release from lockdowns.
		Restarted PrEP during the pandemic. Started PrEP during the pandemic. Stock‐pilling. Stopped PrEP.
2. Sexual behaviour and relationship changes following COVID‐19 restrictions	Changes to sexual activity	Adhering (or not) to guidelines. Advise on dating apps.
	Assessing risks of others	Changing status (on apps). Less opportunity to meet people.
	Use of dating apps	Other behaviour—hand washing, social distancing, LFT testing, etc. Other people's behaviour. Putting others at risk. Sexual behaviour (or changes to).
3. NHS service provision during the COVID‐19 pandemic	Access to clinic, appointments and PrEP	Access to (or lack of) PrEP. Advice and information on PrEP during COVID (or lack of it).
		Getting a test during lockdown. Lack of access to clinics/appointments.
	Information from healthcare professionals STI testing	Limited resources.
4. Wider contextual effects of the COVID‐19 pandemic	Impact on STI transmission Impact on mental health and well‐being Long‐lasting behavioural changes	Change in routine. Community response to COVID‐19 and lockdown. Giving the body a break from PrEP. Increase in barrier methods (or lack of). Information through relatives and friends. Lasting changes to behaviours (or lack of). Less infections circulating. Link between COVID‐19 and sexual health. News around protection.

### 3.1 Theme 1: PrEP Use During the COVID‐19 Pandemic

#### 3.1.1 Stopping and Restarting PrEP

Most participants who were current PrEP users stopped taking PrEP during the pandemic largely due to changes in sexual activity, such as reduced sexual activity or a change in relationship status or number of sexual partners. A reduction in sexual activity was not the only reason for stopping PrEP during the pandemic. Access to PrEP was also a key factor. As PrEP services were disrupted, participants were unable to access PrEP.

The only time that I've not been able to take it (PrEP) was when obviously the virus and everything shut down. I wasn't able to get a supply for about a month and a half, two months?

(P4, DO‐PrEP, 30–39 years, end of first lockdown, July 2020)

However, many participants described that they were unsure whether they could safely stop and restart PrEP. Participants described nervousness around stopping suddenly and were unaware of whether this may cause unwanted side effects.

I was scared to stop taking it because I know that I was told to take it, so, and was I able to stop? I actually did speak to the clinic, last week when I phoned up and said, ‘could I have stopped it?’, ‘oh yeah, yeah, just start it up if you need to’, but I carried on, I just did it as I initially thought, because I had no way of contacting the clinic because of them being shut or busy or whatever.

(P2, DO‐PrEP, 40–49 years, during first lockdown May 2020)

Information circulating on news and media sites was reported to play a part in decisions on PrEP use. One participant continued to take PrEP during the pandemic, as he saw misinformation in a news article that PrEP could protect against coronavirus.

They had trials then, I think it was Italy or Spain, they had trials there, half the people were given PrEP and other were given medication if you got HIV, to see if it can fight this coronavirus, so I thought that makes sense, so I went back on PrEP, just in case.

(P7, DO‐PrEP, 50–59 years, during first lockdown, May 2020)

For some, establishing and maintaining a PrEP routine was seen as key to maintaining normality during the pandemic. Participants described uncertainty around when restrictions would be lifted and resuming sexual activity.

But I still carried on taking PrEP, because it was part of my daily routine.

P34, DO‐PrEP, 40‐49 years, 2nd ‘fire breaker’ lockdown [short lockdown of about two weeks to alleviate pressure on health services] Oct 2020

#### 3.1.2 Adopting Flexible Approaches to PrEP

A switch to event‐based PrEP, or a more flexible approach to PrEP use, was generally adopted. Some cited advice received from PrEP services and healthcare professionals (HCPs) on this, whereas others saw it in the media. This advice was perceived positively among participants; the service suggested an alternative to PrEP use rather than not providing information regarding the matter, especially in the uncertain period that the pandemic brought.

I had a text message because I had an appointment due in April (2020) so at the height of lockdown, it was like a leaflet texted to me suggesting event‐based dosing.

(P24, UPrEP, 18–29 years, end of first lockdown August 2020)

### 3.2 Theme 2: Sexual Behaviour and Relationship Changes Following COVID‐19 Restrictions

#### 3.2.1 Changes to Sexual Activity

Sexual activity was impacted by pandemic restrictions; little to no sexual activity was reported by the majority. Some participants reported that they did not strictly adhere to the Government's COVID‐19 pandemic restrictions (national and local lockdowns and other social restrictions) and felt they had to continue to meet people for their mental well‐being.

Even though you know it … the risks of COVID. I need that [sexual] contact cos I live alone. Yeah. It has reduced greatly but I still need that contact.

(P1, DO‐PrEP, 40–49 years, end of first lockdown July 2020)

Other participants felt strongly about the importance of adhering to guidelines and not meeting people, as it was perceived to be engaging in behaviour that could endanger them and others. This was an attitude which became more relaxed as society went into later lockdowns.

At the start of lockdown, the first one in March [2020], I feel like it was just like the general consensus that most people weren't meeting. But we're in a full lockdown now and I don't think people have the same attitude, and I feel like people are a lot more unreserved about meeting. I mean, myself included.

(P16, UPrEP, 18–29 years, winter lockdown, January 2021)

#### 3.2.2 Assessing the Risks of Others

One participant linked behaviour related to COVID‐19 and behaviour relating to sexual health, describing his unwillingness to meet people who did not adhere to COVID guidelines, as he believed that they may be more likely to take risks in general and therefore more likely to have an STI.

I still get people messaging wanting to meet up and sort of okay, that was a shame because they always used to be fun, but I'm not going to be meeting up with them now. Not just now, but I probably won't meet up with them afterwards, because as far as I can see they're just taking stupid risks. And if they're going to take risks like that now, then they're taking risks like that all the time.

(P56, DO‐PrEP, 40–49 years, Autumn restrictions, November 2020)

#### 3.2.3 Use of Dating Apps

Participants referenced advice from social networking or online dating apps, such as Grindr, which advised people to stay at home and not meet others, and the ability for individuals on dating sites to change their status to display whether they were willing to meet.

I think it's definitely been the case for like there's been so many more things on Grindr and everything to say stay home and don't meet up with people obviously.

(P25, DO‐PrEP, 18–29 years, end of first lockdown August 2020)

Relationship changes were evident among participants secondary to pandemic restrictions —some participants moved in with their partners, and some participants reported a change from polygamous to monogamous relationships. As a result, there were reports of stopping PrEP indefinitely.

Long story short, one of my best friends, has now become my boyfriend. We've been living together and that was really the only [sexual] activity I was having.

(P54, DO‐PrEP, 18–29 years, end of first lockdown, September 2020)

### 3.3 Theme 3: NHS Service Provision During the COVID‐19 Pandemic

#### 3.3.1 Access to Clinics

As a result of pandemic restrictions, PrEP clinics saw disruption across all health boards, and many participants experienced appointment cancellations. Some clinics cancelled appointments with no rescheduling, making it difficult for participants to receive another supply of PrEP.

with the whole COVID thing they stopped taking appointments at the PrEP clinic, which was part of the reason why I've stopped taking it now.

(P8, DO‐PrEP, 18–29 years, first lockdown, May 2020)

However, health boards differed in their approach. Participants reported that clinics used approaches such as contacting patients as soon as possible to schedule an appointment, accessing services over the phone and providing a usual supply of PrEP.

Then they rang me back and said, ‘Oh we're gonna try and open the clinic the following week, would you like to book an appointment, a new appointment?’

(P54, DO‐PrEP, 18–29 years, end of first)

Some participants reported that they felt it important to receive a supply of PrEP from a consistent source and that they are familiar with and could trust. Participants reported friends buying PrEP online, which added to concerns about whether the medication was ‘proper pills’.

A lot of people, they didn't get the message. Although they're on the pills, they didn't receive their emails. They didn't know they were … and … they got so frustrated that they weren't getting their proper pills that they went out and bought them.

(P1, DO‐PrEP, 40–49 years, end of first lockdown July 2020)

#### 3.3.2 Advice From HCPs

Perceptions of support during the pandemic from PrEP services differed among participants. Participants described the importance of having individualised support—as not all patients will require the same amount of support.

They were kind of very open, they said have you got any questions, and I definitely sat there for about a minute, and said oh actually, could I just check this, could I check this, and then they'd sit back down, and they'd go through it with me again, for like another five, 10 minutes, just to put any doubt out of my mind.

(P15, DO‐PrEP, 18–29 years, end of first lockdown, August 2020)

#### 3.3.3 STI Testing

The rollout of Sexual Health Wales (previously called Frisky Wales) NHS postal testing service in May 2020 meant that participants were able to continue to do STI testing without attending clinics. This was perceived by participants as convenient and efficient without the requirement of a clinic visit and a consultation (‘the hard stuff’), suggesting that they are more likely to undertake regular testing.

And I'm trying to think of positive things have come out of … I think, you know, the rollout of Frisky Wales, is amazing. I think that has definitely been seen now as kind of like absolving responsibility of the hard stuff which has to be … like I say, the conversations.

(P5, UPrEP, 18–29 years, week before firebreak lockdown, October 2020)

### 3.4 Theme 4: Wider Contextual Effects of the COVID‐19 Pandemic

#### 3.4.1 Impact on STI Transmission

Participants viewed the pandemic as a beneficial period for reducing STI transmissions, with one individual referring to it as an ‘STI firebreak’ (P8, UPrEP). However, this reduction was not intentional. The primary concern was the fear of contracting COVID‐19, which inadvertently led to fewer STIs being transmitted.

But I don't think that's from a standpoint of trying to limit STIs it's just purely because of COVID.  So yeah, I think … yeah.  Like somewhat in terms of culturally reducing their circle, but not so much in terms of the intent of not meeting people is to avoid STIs.

(P10, UPrEP, 18–29 years, Autumn restrictions, November 2020)

#### 3.4.2 Impact on Mental Health and Well‐Being

Several participants also talked about how the pandemic and the consequent lack of social opportunities had negatively impacted their mental health and well‐being. Sex was reported to be a fundamental part of lives, and some participants reported feeling lonely and lost.

When this virus came in, I didn't meet no one at all, I stopped meeting people [pause] massive, that's something I had to live with, and I had to deal with.

(P7, DO‐PrEP, first lockdown, May 2020)

#### 3.4.3 Long‐Lasting Behavioural Changes

Some participants explained that the pandemic was bound to have a lasting impact on people's willingness to meet strangers and be more cautious before engaging in sexual behaviours.

I hope people in general will have more of a thought before they do certain things.

(P12, UPrEP, 18–29 years, winter lockdown, December 2020)

However, the majority reported that they expected people to increase their sexual activity and the number of sexual partners in the post‐pandemic period, as they believed that people have been deprived of physical contact for an extended period leading to ‘I don't care’ attitudes and an increase in willingness to engage in sexual activity. Some reflected concerns that there may be a strain on sexual health services during immediate release from lockdown.

And like after lockdown ends, I think it's going to go sky high because people have been deprived of that lifestyle for so long that I think it's just going to kind of go whoosh.

(P18, UPrEP, 18–29 years, winter lockdown, January 2021)

## 4 Discussion

In Wales, during the COVID‐19 pandemic, PrEP use generally followed a similar pattern to sexual activity—during periods of low sexual activity, PrEP use was reported as minimal. However, some individuals reported continued PrEP use, regardless of the lockdowns and changes in personal circumstances. There was a focus on flexibility in the use of PrEP, such as an event‐based approach. NHS service restrictions impacted participants' access to services, leading to feelings of confusion and a lack of support for PrEP use. Additionally, the perceived importance of having a credible and reliable source of PrEP was highlighted among participants, alongside the idea of having personalised support. It was suggested that the pandemic would have a lasting impact on sexual behaviour and STI testing. Finally, there have been some perceived benefits to the COVID‐19 pandemic, including the accelerated rollout of postal STI testing, changes to relationship statuses and the possibility of a ‘firebreak’ reduction in STI transmission. The findings must be taken in context with the timings of interviews as some participants interviewed in earlier lockdowns had stronger views about adhering to social restrictions than those interviewed in later lockdowns. For MSM, there is a historical relationship with pandemics for those who lived through the HIV crisis, and so this may have impacted their response to the COVID‐19 pandemic. This could be explored in more detail in future work.

Survey studies in the United Kingdom and elsewhere have similarly reported a reduction in sexual activity in MSM during COVID‐19 with fewer opportunities to engage in sexual activity, similar to our findings [12, 19, 20]. Surveys have also shown that isolation and lack of sexual contact during COVID‐19 were associated with poor mental health for people living with HIV [21]. Previous research has also indicated that COVID‐19 has resulted in challenges that MSM experience accessing sexual health clinics, thereby impacting PrEP adherence [10, 22, 23, 24] and mental health [25, 26, 27]. Participants in our secondary analysis study also described mental health issues as a result of restrictions; however, there was not a focus on mental health unless disclosed by the participant. Less research has been conducted exploring the context of these changes and impacts using qualitative methods, although some interesting studies have also identified PrEP adherence [23] and lost community connections [28] to be problematic for MSM during the COVID‐19 pandemic. Issues of lockdown fatigue have also been identified in our own work [11]. PrEP services in Wales have undergone major reform since the beginning of the COVID‐19 pandemic [29]; however, there appear to be differences in how these are implemented between Health Boards in Wales. Service improvement initiatives have been developed including a PrEP self‐referral pathway using the Sexual Health Wales online home test and post‐service, and an app that is designed to be used by the individual to keep track of their PrEP use. Telemedicine included virtual consultations and no‐contact prescription collection. In addition, the feasibility and acceptability of accessing PrEP via community pharmacies is being explored. Future research should look at PrEP users' views of these approaches to telemedicine and enhanced community access to PrEP.

To our knowledge, this is one of the first qualitative interview studies exploring the experiences of MSM PrEP users during the COVID‐19 pandemic. Previous studies have examined the impact of the COVID‐19 pandemic on MSM, most of which have been survey studies. Our qualitative design allowed for a detail‐rich description of experiences and feelings, which may not be as well conveyed through survey responses. However, our study has focused on a specific sub‐population—white MSM who have accessed PrEP in Wales. Our findings are of relevance to places where the majority of PrEP users are MSM (as in Wales) but may have less applicability in other settings. Another strength of this study is the wide age range of participants. Although remote interviews may have facilitated the recruitment of individuals from across Wales including rural areas, we may have excluded individuals with no access to the required technology or Internet. A potential bias may arise from using data sets to answer a different research question. Neither study specifically included the topic of COVID‐19; data were included only if a participant discussed it. Ultimately, there may be differences in views among the participants who did not discuss the pandemic. We are aware that this will affect the transferability of these findings. However, 78% of potential interviews were included in this secondary analysis.

Our research indicates that targeted interventions should be implemented to give PrEP users sufficient support with PrEP use, so they have accurate information on PrEP use such as stopping/re‐starting and can be sign‐posted to relevant professionals for their information needs. This may be implemented by creating personalised care plans for PrEP users and creating a PrEP service that is adopted nationally, mitigating the differences in support and access to care between health boards. This will be important if similar restrictions on access to sexual health clinics are implemented in the future, especially as the delivery of PrEP continues to extend. Further research should be conducted on more flexible approaches to PrEP for other groups, so the disparity of inequalities with regard to HIV prevention is not widened; especially when other individuals who are at risk of HIV acquisition, such as female sexworkers, are already faced with the burden of health inequalities. Furthermore, more emphasis on different approaches to STI and HIV testing, such as postal testing, through multimedia campaigns will be pivotal in reducing transmissions. In Wales, this will be essential to achieve zero HIV transmission by 2030 as per the HIV Action Plan for Wales 2023–2026 [29].

## 5 Conclusion

In conclusion, this study offers personal experiences on PrEP use and access and changes in sexual behaviour during the COVID‐19 pandemic. It also highlights the importance of maintained and consistent support from relevant bodies during times when restrictions are imposed and access to services is atypical. As PrEP services are expanding, these findings can be utilised in implementing interventions that best achieve patient‐centred care.

## Author Contributions

**Zahraa Khammas:** writing–original draft, formal analysis. **David Gillespie:** conceptualisation, funding acquisition, writing–review and editing, methodology, supervision, data curation, project administration, investigation, resources. **Adam Dale Newman Williams:** conceptualisation, methodology, data curation, supervision, project administration, writing–review and editing, investigation, formal analysis, resources, validation. **Jane Nicholls:** investigation, supervision, formal analysis, writing–review and editing. **Fiona Wood:** investigation, funding acquisition, validation, formal analysis, supervision, resources, data curation, writing–review and editing, software.

## Ethics Statement

DO‐PrEP was reviewed and approved by the Wales Research Ethics Committee 3 (reference number 19/WA/0175). The qualitative sub‐study of UPrEP was reviewed and approved by the Cardiff University School of Medicine Ethics Committee (reference number 20/21).

## Conflicts of Interest

D.G., A.D.N.W. and J.N. report receiving funding from Health and Care Research Wales during the conduct of this study. D.G., A.D.N.W. and J.N. are on the steering committee for the Fast Track Cardiff & Vale group. This group is a local branch of the Fast Track Cities initiative aiming at eradicating HIV by 2030. J.N. has received grants and from Gilead Sciences Ltd. The other authors declare no conflicts of interest.

## Data Availability

Data (thematic coding matrices) are available from the authors on request. Due to privacy/ethical restrictions, other data (e.g., full transcripts) are not available.
